# ABO Blood Group and Biomarker-Based Risk in Acute Pulmonary Embolism: A Retrospective Cohort Study

**DOI:** 10.3390/jcm15041432

**Published:** 2026-02-12

**Authors:** Abdulkader Jamal Eddin, Stefan Iulian Stanciugelu, Arnaldo Dario Damian, Bogdan Petru Miutescu, Oana Elena Tunea, Ioana Monica Mozos

**Affiliations:** 1 Doctoral School, “Victor Babeş” University of Medicine and Pharmacy, 300041 Timişoara, Romania; jamal_eddin6@yahoo.com; 2 Center for Translational Research and Systems Medicine, “Victor Babeş” University of Medicine and Pharmacy, 300173 Timişoara, Romania; ioanamozos@umft.ro; 3 Gastroenterology and Hepatology Clinic, “Pius Brînzeu” County Emergency Clinical Hospital, 300723 Timişoara, Romania; 4 Orthopedics II Research Center, “Pius Brînzeu” County Emergency Clinical Hospital, 300723 Timişoara, Romania; 5 Orthopedics Clinic II, “Pius Brînzeu” County Emergency Clinical Hospital, 300723 Timişoara, Romania; 6 Neurology Clinic II, “Pius Brînzeu” County Emergency Clinical Hospital, 300723 Timişoara, Romania; dariodamian996@gmail.com; 7 Division of Gastroenterology and Hepatology, Department of Internal Medicine II, “Victor Babeş” University of Medicine and Pharmacy, 300041 Timişoara, Romania; 8 Advanced Regional Research Center in Gastroenterology and Hepatology, “Victor Babeş” University of Medicine and Pharmacy, 300041 Timişoara, Romania; 9 Advanced Research Center in Cardiovascular Pathology and Hemostaseology, “Victor Babeş” University of Medicine and Pharmacy, 300173 Timişoara, Romania; ancusa.oana@umft.ro; 10 Internal Medicine Clinic, Emergency Municipal Clinical Hospital, 300041 Timişoara, Romania; 11 Department of Functional Sciences-Pathophysiology, “Victor Babeş” University of Medicine and Pharmacy, 300173 Timişoara, Romania

**Keywords:** pulmonary embolism, ABO blood group, biomarkers, NT-proBNP, CRP, procalcitonin, D-dimer, CK-MB, sepsis, venous thromboembolism

## Abstract

**Background**. Non-O ABO blood groups are known to confer an increased risk of venous thromboembolism, primarily through higher circulating levels of von Willebrand factor and factor VIII. However, it remains unclear whether ABO type affects biochemical profiles at the time of presentation or alters the prognostic value of commonly used biomarkers in acute pulmonary embolism (PE). This study examined the relationship between ABO blood group, baseline biomarkers, and short-term clinical outcomes in patients with confirmed acute PE. **Methods**. We performed a retrospective cohort study of adults admitted with computed tomography pulmonary angiography-verified PE at a single tertiary center. Associations between biomarkers and clinical outcomes were assessed using logistic regression adjusted for age, sex, active cancer, chronic kidney disease, obesity, and ABO group. Interaction terms tested whether ABO type modified biomarker–outcome relationships. **Results**. Among 317 included patients (median age 69 years), in-hospital mortality was 11.0%; 29.6% experienced severe PE, 48.3% developed infection, and 11.7% developed sepsis. Baseline biomarker distributions were similar across ABO groups, and multivariable models showed no independent association between non-O type and biomarker levels. NT-proBNP, CRP, and procalcitonin predicted in-hospital mortality, while NT-proBNP, procalcitonin, and CK-MB predicted severe PE. CRP, procalcitonin, D-dimer, creatinine, and leukocyte count were associated with infectious and septic complications. ABO type did not meaningfully modify biomarker–outcome relationships, aside from one exploratory interaction for infection. Sensitivity analyses confirmed the robustness of these findings. **Conclusions**. ABO blood group did not influence baseline biomarker profiles or the prognostic performance of key biomarkers in acute PE. Early outcomes were instead driven by indicators of right ventricular strain, inflammation, and renal dysfunction.

## 1. Introduction

ABO blood group is a key genetic determinant of venous thromboembolism (VTE) risk [1,2,3,4,5,6,7,8,9,10]. Individuals with non-O blood types have consistently been shown to exhibit higher circulating levels of von Willebrand factor (vWF) and factor VIII due to reduced clearance of vWF multimers [1,2,3,4,5,6,7,8,9]. These hemostatic differences promote a prothrombotic environment, contributing to the increased incidence of deep vein thrombosis and pulmonary embolism (PE) observed in non-O individuals [2,3,10,11,12]. Recent large-scale genetic studies have further refined the association between the ABO locus and venous thromboembolism risk, demonstrating that multiple non-O haplotypes confer a modest but consistent increase in both incident and recurrent VTE, with important population-specific differences in haplotype structure and effect size across ancestries [13]. Beyond coagulation pathways, ABO antigens influence endothelial function, inflammation, and vascular adhesion, raising the possibility that inherited blood group characteristics may interact with the biological response to acute PE [10,14,15,16]. However, whether these chronic ABO-mediated differences have clinical relevance after an embolic event has already occurred remains uncertain.

Beyond its established role in venous thromboembolism susceptibility, ABO blood group has been implicated in a broader range of clinical phenotypes, including hemorrhagic complications and infectious diseases. Non-O blood groups are associated with higher circulating levels of von Willebrand factor and factor VIII, conferring increased thrombotic risk, whereas blood group O has been linked to a relatively higher bleeding tendency, particularly in settings of anticoagulation or trauma. In addition, ABO antigens expressed on endothelial and epithelial surfaces may influence inflammatory signaling and host–pathogen interactions, with reported associations between ABO type and susceptibility or severity of selected infections. Together, these observations suggest that ABO blood group may modulate both hemostatic balance and systemic inflammatory responses. However, whether such inherited differences meaningfully influence biomarker profiles or short-term prognosis once acute pulmonary embolism has occurred remains uncertain [2,3,17].

Early risk assessment in acute PE is critical and relies on a structured integration of clinical, imaging, and biochemical markers [18,19,20,21,22]. The Pulmonary Embolism Severity Index (PESI) and simplified PESI (sPESI) stratify early mortality risk based on age, comorbidities, vital signs, and clinical findings, forming the basis of major guideline recommendations. The European Society of Cardiology (ESC) algorithm further incorporates imaging evidence of right ventricular (RV) dysfunction, reflecting acute pressure overload, and cardiac biomarkers such as troponin and N-terminal pro-B-type natriuretic peptide (NT-proBNP) to identify intermediate- and high-risk patients. These biomarkers capture the hemodynamic consequences of PE, while inflammatory and metabolic markers such as C-reactive protein (CRP), procalcitonin, creatinine, and D-dimer reflect systemic stress, organ dysfunction, infection, or ongoing thrombus turnover [20,22,23,24,25,26,27,28].

Although ABO blood group influences hemostatic and inflammatory pathways at baseline [3,4,6,12,29,30,31], it is unknown whether these inherited differences translate into distinct biomarker patterns at the time of PE presentation, or whether ABO status modifies the prognostic utility of biomarkers routinely used in clinical risk assessment. Clarifying this relationship is clinically relevant. If ABO-mediated biological variation influences biomarker interpretation, risk stratification tools may perform differently across blood group categories. Conversely, if acute pathophysiological processes dominate the biochemical landscape, ABO type may be irrelevant for short-term prognosis.

This study therefore addressed two questions in a cohort with confirmed acute PE by computed tomography pulmonary angiography (CTPA): (1) whether ABO blood group is associated with differences in baseline biomarker profiles at presentation, and (2) whether ABO type modifies the prognostic associations between key biomarkers, including NT-proBNP, CRP, procalcitonin, creatinine, and D-dimer, and short-term clinical outcomes, such as in-hospital mortality, severe PE manifestations, infection, and sepsis. By examining the interplay between inherited ABO characteristics and acute biomarker responses, we sought to clarify the biological and clinical relevance of ABO type in early PE risk stratification.

## 2. Materials and Methods

### 2.1. Study Design and Setting

This retrospective observational study was conducted at the Municipal Hospital Timișoara, Romania. The analysis used the institutional database of adult patients admitted with acute pulmonary embolism (PE). The dataset contained demographic, clinical, imaging, laboratory, and in-hospital outcome information collected at presentation or throughout the first 24 h of hospitalization. This study represents a predefined secondary analysis designed to examine the relationships between ABO blood group, baseline biomarker profiles, and short-term clinical outcomes in acute PE.

### 2.2. Study Population

Eligible participants were adults (≥18 years) with a confirmed diagnosis of acute PE based on computed tomography pulmonary angiography (CTPA). Patients were excluded if ABO blood group was unavailable, if baseline laboratory values at presentation were missing, or if in-hospital outcome data were incomplete.

### 2.3. Baseline Clinical Characteristics

Demographic variables (age, sex, place of residence), comorbidities (hypertension, chronic obstructive pulmonary disease, atrial fibrillation, chronic kidney disease, active cancer, prior stroke, heart failure, myocardial infarction, asthma, dementia, chronic venous insufficiency, diabetes mellitus, pulmonary hypertension, hematologic disease, obesity) and the Pulmonary Embolism Severity Index (PESI) score and class were recorded at admission. Prior antiplatelet and anticoagulation therapy were also documented.

### 2.4. ABO Blood Group Determination

ABO blood type was determined through routine admission serology using standard agglutination techniques. The primary exposure was dichotomized as O versus non-O (A, B, or AB). A secondary four-category variable (A/B/AB/O) was used for descriptive analyses.

### 2.5. Biomarkers

All laboratory biomarkers obtained at presentation were included. Biomarkers were grouped into functional categories:

- Hematologic: leukocyte, lymphocyte, and neutrophil counts; platelet counts
- Renal/metabolic: creatinine, glucose, sodium, potassium
- Hepatic: total and direct bilirubin, aspartate aminotransferase (AST), alanine aminotransferase (ALT), gamma-glutamyl transferase (GGT), total serum proteins
- Inflammatory: C-reactive protein (CRP), erythrocyte sedimentation rate, procalcitonin
- Cardiac strain/injury: N-terminal pro-B-type natriuretic peptide (NT-proBNP), creatine kinase-MB
- Coagulation: D-dimer, fibrinogen, prothrombin time, activated partial thromboplastin time (aPTT), international normalized ratio (INR)
- Lipid profile: total cholesterol, low-density lipoprotein (LDL), high-density lipoprotein (HDL), triglycerides

Units were standardized according to the hospital laboratory system. Biomarkers with more than 30% missingness (e.g., ferritin, calcium) were excluded from primary regression analyses but retained for descriptive reporting.

### 2.6. Clinical Outcomes

The primary outcome was in-hospital mortality from any cause. Secondary outcomes included a composite severe PE endpoint, defined as the occurrence of in-hospital death, requirement of systemic thrombolysis, requirement of invasive mechanical ventilation or non-invasive positive pressure ventilation (CPAP). Additional evaluated outcomes during hospitalization were ischemic stroke confirmed by imaging, clinically diagnosed infections and sepsis, identified based on physician documentation and systemic inflammatory criteria. All outcomes were obtained directly from the medical record.

### 2.7. Ethical Considerations

The study was approved by the Ethics Committee of the Municipal Hospital Timișoara. All procedures adhered to the Declaration of Helsinki and applicable national regulations. Owing to the retrospective design and use of anonymized data, the requirement for informed consent was waived.

### 2.8. Data Preprocessing and Transformations

Continuous variables were assessed for distributional characteristics. Biomarkers with right-skewed distributions (for example, D-dimer, CRP, NT-proBNP, creatinine, triglycerides, creatine kinase-MB, erythrocyte sedimentation rate, and procalcitonin) were natural-log transformed prior to modeling. For linear models, regression coefficients for continuous biomarkers were exponentiated and expressed as geometric mean ratios (GMRs), which can be interpreted as percent differences in biomarker levels. For logistic models, regression coefficients are reported as odds ratios per 1 standard deviation increase in log-transformed biomarker values. Missing data were handled through complete-case analysis, and the number of observations is reported for each model. No imputation was performed. Laboratory variables available in fewer than approximately 70% of patients (e.g., ferritin, erythrocyte sedimentation rate) were excluded from primary regression analyses, while remaining analyses were conducted using complete-case data.

### 2.9. Statistical Analysis

Descriptive analyses. Categorical variables were compared between ABO groups using χ^2^ or Fisher’s exact tests, as appropriate. Continuous variables were compared using ANOVA or the Kruskal–Wallis test, depending on distribution. Biomarker distributions were summarized using medians and interquartile ranges.

Association between ABO group and baseline biomarkers. Multivariable linear regression models were fitted for each log-transformed biomarker as the dependent variable. The primary predictor was non-O versus O blood group. All models were adjusted for age, sex, active cancer, chronic kidney disease, and obesity. Results are presented as adjusted geometric mean ratios (GMRs) comparing non-O with O, derived from exponentiated regression coefficients. A GMR of 1 indicates no difference, whereas values > 1 or <1 indicate higher or lower levels, respectively. The number of patients included (N) reflects complete-case availability for each biomarker.

Biomarkers as predictors of clinical outcomes. Separate multivariable logistic regression models were constructed for in-hospital mortality, the composite severe PE endpoint, infection, sepsis, and stroke (when the number of events permitted analysis). Each model included one biomarker at a time and was adjusted for age, sex, active cancer, chronic kidney disease, obesity, and ABO blood group (non-O vs. O). Results are presented as adjusted odds ratios per 1 standard deviation increase in log-transformed biomarker values, with 95% confidence intervals.

Effect modification by ABO. To evaluate whether ABO blood group modified the prognostic association between biomarkers and clinical outcomes, interaction terms of the form “log(biomarker) × non-O” were added to the multivariable logistic models. Interaction models were fitted for in-hospital mortality, the composite severe PE endpoint, infection, and sepsis. Analyses were performed for all available biomarkers. When interaction terms were non-significant, ABO-stratified predicted probabilities were examined descriptively.

Power/precision analysis. Given the retrospective design and fixed sample size, we performed a minimal detectable effect size (MDES) analysis for the primary exposure comparison (O vs. non-O). Assuming a two-sided α = 0.05 and 80% power with the observed group sizes (O *n* = 69; non-O *n* = 248), the study was powered to detect odds ratios (non-O vs. O) of approximately ≥2.55 (or ≤0.39) for in-hospital mortality, ≥2.19 (or ≤0.46) for the composite severe PE endpoint, ≥2.27 (or ≤0.44) for in-hospital infection, and ≥2.55 (or ≤0.39) for in-hospital sepsis. For rare outcomes such as ischemic stroke (*n* = 14 events), only very large effects would be detectable (MDES OR ≈ 3.5); therefore, rare-event and interaction analyses should be interpreted as exploratory.

Sensitivity analyses. Sensitivity analyses included (1) modeling ABO blood type using all four categories (A/B/AB/O), (2) repeating regression models after excluding patients with active cancer, and (3) repeating analyses after excluding biomarker values above the 99th percentile.

Software. All analyses were conducted in Python (3.14.0) using the pandas, numpy, and statsmodels libraries.

## 3. Results

A total of 317 patients with acute pulmonary embolism were included in the analysis. The distribution of ABO blood groups together with demographic and clinical characteristics by ABO group are shown in Table 1.

In-hospital outcomes stratified by ABO group (O, A, B, AB) are presented in Table 2.

Median baseline biomarker concentrations for each ABO category are presented in Table 3.

Across biomarkers, unadjusted comparisons showed no clinically meaningful differences between ABO groups, as presented in Table 4. NT-proBNP and D-dimer showed wide variability in all groups, reflecting heterogeneous disease severity at presentation, without a consistent pattern across ABO categories.

Non-O blood group was not independently associated with higher biomarker concentrations at presentation. GMRs ranged from 0.80 to 1.40, and all 95% confidence intervals included 1 with non-significant *p* values. These findings indicate that ABO blood type did not confer a distinct biochemical phenotype in acute PE.

### 3.1. Biomarkers as Predictors of In-Hospital Outcomes

Adjusted odds ratios (ORs) per 1 SD increase are summarized in Table 5 and further detailed by each specific outcome in related Table 6, Table 7, Table 8 and Table 9.

The number of ischemic stroke events (*n* = 14) was insufficient for reliable multivariable modeling, therefore adjusted biomarker–stroke associations were not examined.

Among all biomarkers evaluated, NT-proBNP, procalcitonin, and CRP were independent predictors of in-hospital death. Higher levels of these biomarkers at presentation were associated with substantially increased mortality risk. Creatinine, CK-MB, leukocytes, D-dimer, and platelet count were not statistically significant predictors after full adjustment. These findings indicate that early cardiac strain and systemic inflammatory activation are the strongest biochemical indicators of short-term mortality in acute PE.

Several biomarkers demonstrated significant associations with the composite outcome: NT-proBNP, procalcitonin and CK-MB. CRP showed a borderline association. Creatinine and D-dimer were not significant predictors. Leukocyte count was inversely associated with the composite endpoint, suggesting lower composite risk at higher leukocyte levels. Although consistent with unadjusted trends in the dataset, this counterintuitive association likely reflects confounding by clinical presentation and is addressed in the Discussion.

Overall, these findings suggest that cardiac strain, systemic inflammation, and early microbial activation (as captured by procalcitonin) are important determinants of severe clinical deterioration.

CRP, procalcitonin, D-dimer, and leukocyte count were independently associated with higher infection risk. NT-proBNP, CK-MB, creatinine, and platelet count were not strongly associated with infection.

Stronger associations were observed for sepsis: CRP, procalcitonin, NT-proBNP, creatinine, and D-dimer were all independent predictors. Taken together, these analyses indicate that inflammatory activation (CRP), microbial response pathways (procalcitonin), cardiac strain (NT-proBNP), and early renal impairment (creatinine) are strong predictors of infectious and septic complications, whereas D-dimer contributes modest additional prognostic information.

**Figure 1:**
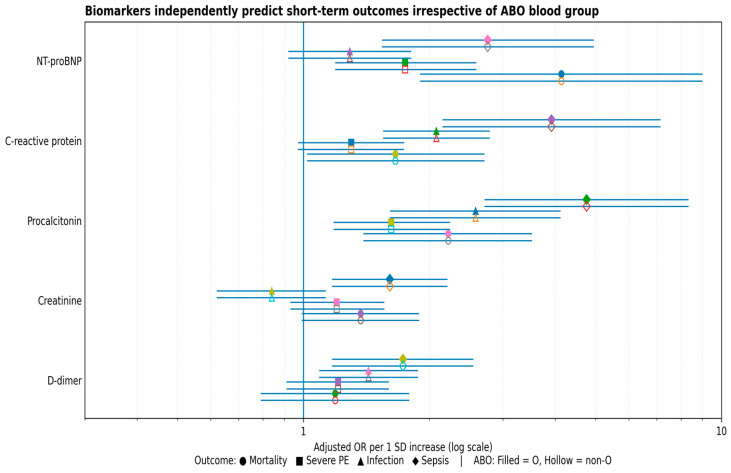
Biomarkers independently predict short-term clinical outcomes irrespective of ABO blood group.

Forest plot (Figure 1) showing adjusted odds ratios (ORs) per 1-SD increase in NT-proBNP, C-reactive protein, procalcitonin, creatinine, and D-dimer for in-hospital mortality, severe pulmonary embolism, infection, and sepsis. Filled symbols indicate blood group O and hollow symbols indicate non-O blood groups. The overlap of effect estimates across ABO strata illustrates the absence of meaningful effect modification by ABO blood group.

### 3.2. Effect Modification by ABO Blood Group

NT-proBNP was selected as the primary biomarker for interaction testing because it showed the strongest and most consistent association with adverse outcomes, particularly in-hospital mortality. Interaction analyses were restricted to patients with available NT-proBNP and complete covariate data. For in-hospital mortality, the NT-proBNP × ABO interaction was not statistically significant (*p* for interaction = 0.63), indicating that the association between NT-proBNP and death did not differ meaningfully between O and non-O patients. For the composite severe PE endpoint and for sepsis, interaction terms were likewise non-significant (*p* for interaction = 0.429 and *p* = 0.192, respectively), although confidence intervals for the O group were wide due to small event numbers. In contrast, a statistically significant interaction was observed for in-hospital infection (*p* for interaction = 0.034), with NT-proBNP showing a numerically stronger association with subsequent infection in O patients than in non-O patients. Given the limited number of events and the wide confidence intervals, this finding should be interpreted as exploratory.

Exploratory interaction analyses for the all other biomarkers did not reveal meaningful evidence of effect modification by ABO blood group. Overall, these results suggest that ABO type does not materially alter the prognostic utility of NT-proBNP or other key biomarkers in acute PE.

### 3.3. Sensitivity Analyses

Modeling ABO blood type using four categories (A/B/AB/O) instead of a dichotomous non-O versus O variable did not materially alter the associations between ABO group, biomarkers, and clinical outcomes. Excluding patients with active cancer yielded effect estimates that were similar in magnitude and direction to the main analyses. Likewise, repeating the biomarker–outcome models after excluding observations above the 99th percentile of each biomarker distribution did not meaningfully change the results. Therefore, only the primary analyses are presented in detail.

## 4. Discussion

In this cohort of 317 patients with acute pulmonary embolism, ABO blood group was not associated with differences in baseline biomarker concentrations and did not materially modify the prognostic value of NT-proBNP or other key biomarkers. NT-proBNP emerged as a strong independent predictor of in-hospital mortality, while CRP, procalcitonin, creatinine, and D-dimer were independently associated with severe PE manifestations and with infectious and septic complications. Baseline levels of NT-proBNP, D-dimer, CRP, creatinine, procalcitonin, leukocytes, and platelets were broadly similar across ABO categories, and multivariable models showed no independent association between non-O blood type and biomarker profiles. Interaction analyses likewise provided no consistent evidence that ABO group altered biomarker–outcome relationships. NT-proBNP, CRP, and procalcitonin independently predicted in-hospital mortality. NT-proBNP, procalcitonin, and CK-MB predicted the composite severe PE outcome, and inflammatory and renal markers showed robust associations with infection and sepsis. These findings highlight the central prognostic roles of right ventricular strain, systemic inflammation, and renal dysfunction in acute PE.

Previous studies have primarily linked ABO blood group to incident VTE risk, largely through von Willebrand factor and factor VIII pathways [4,5,8,9,18,19,20,21,31,32,33,34]. Evidence regarding its influence on acute PE outcomes is limited [3,29,30,35,36]. Our results suggest that, once PE occurs, ABO group does not meaningfully affect early biomarker patterns or in-hospital prognosis. By contrast, the strong associations observed for NT-proBNP, CRP, and procalcitonin align with the prior prognostic literature emphasizing the impact of myocardial strain and systemic inflammation [37,38,39,40,41,42,43,44].

The inverse association between leukocyte count and the composite severe PE endpoint, although consistent in our dataset, is not physiologically intuitive and likely reflects confounding by clinical presentation rather than a true protective effect. This warrants cautious interpretation. First, leukocyte elevation often reflects a delayed inflammatory response, patients with hemodynamically severe PE may present earlier in the disease course, before leukocytosis develops, introducing reverse causality. Second, the composite severe PE endpoint is driven primarily by circulatory and respiratory failure rather than systemic inflammation, whereas leukocyte count more strongly tracks infectious or inflammatory complications, as supported by its positive association with infection in our cohort. Third, this inverse association was not observed for in-hospital mortality or sepsis, arguing against a biologically protective effect. Taken together, this finding likely reflects residual confounding and timing effects rather than a true protective role of leukocytosis [21,22,25,27,30,36,42,44,45].

The lack of an ABO effect on baseline biomarkers suggests that acute hemodynamic stress and systemic inflammation dominate the biochemical response to PE, overshadowing chronic ABO-mediated differences in hemostatic factors. Similarly, consistent prognostic performance of NT-proBNP, CRP, creatinine, and D-dimer across ABO categories implies that these biomarkers carry comparable risk information in O and non-O patients [18,19,32,35,41,46]. The isolated interaction signal for infection, with wide confidence intervals, is best considered exploratory.

Overall, these results support a model in which ABO group influences VTE susceptibility but has limited relevance for short-term prognosis after PE. In contrast, biomarkers reflecting RV strain, inflammation, and renal status track closely with clinical severity and early complications.

NT-proBNP, widely used for the diagnosis of heart failure, is also a biomarker of right ventricular stress [47]. In the present study, it was significantly associated with in-hospital mortality, PE severity, and sepsis, and can help identify patients who are at higher risk for complications. When PE and sepsis coexist, NT-proBNP levels may be disproportionately elevated due to the additive effects of RV overload and sepsis-induced myocardial dysfunction [48]. The extremely wide confidence intervals observed for NT-proBNP interaction estimates in the O blood group (Table 10) reflect the limited number of events within this subgroup and are consistent with the MDES analysis, which indicates insufficient power to detect anything other than very large interaction effects. These estimates should therefore be interpreted as exploratory and not as evidence of effect heterogeneity.

CRP is an acute-phase reactant, which increases dramatically in response to pro-inflammatory cytokines produced during acute inflammation [49]. It predicts cardiovascular events and is considered a marker of chronic low-grade inflammation, related to vascular wall biology [49]. In the present study, it was significantly associated with in-hospital mortality, infection, and sepsis in patients with PE. In PE, elevated CRP levels are associated with more extensive pulmonary infarction, indicating greater tissue injury and inflammatory burden, which explains higher early mortality [50]. CRP was also previously associated with a systemic prothrombotic effect and myocardial inflammation and stress, exacerbating RV failure [51,52].

Procalcitonin, the precursor of calcitonin, increases significantly in response to pro-inflammatory stimuli like bacterial infections. Procalcitonin interacts with pulmonary embolism primarily through its role as a marker and mediator of systemic inflammation and endothelial dysfunction [53]. This explains its significant associations with in-hospital mortality, PE severity, infection, and sepsis in the present study.

We believe the strengths of this study are a well-characterized cohort with CTPA-confirmed PE, systematic biomarker measurement, prespecified analytical models, consistent adjustment for key confounders, and predefined sensitivity analyses, all of which support robustness.

The limitations of this study include the single-center design, modest sample size, and limited statistical power for interaction analyses and rare outcomes. Residual confounding is possible, and infection and sepsis classifications relied on clinical documentation. Stroke events were too few for adjusted modeling, and long-term outcomes were not available. A further limitation is the modest sample size (N = 317; O *n* = 69; non-O *n* = 248), which provides adequate power only for moderate-to-large ABO-associated effects (minimal detectable ORs ≈ 2.55 for in-hospital mortality and sepsis, 2.19 for severe PE, and 2.27 for infection) and limits inference for rare outcomes (e.g., ischemic stroke; *n* = 14) and subgroup/interaction analyses. Catheter-directed thrombolysis and mechanical thrombectomy were not systematically recorded in the available dataset and could therefore not be analyzed.

These findings do not support ABO blood group as a prognostic factor in acute PE. Risk assessment should continue to rely on established clinical variables and biomarkers such as NT-proBNP, CRP, procalcitonin, creatinine, and D-dimer, which showed clear associations with mortality, severe PE, infection, and sepsis. Larger multicenter studies with standardized outcome adjudication and longer follow-up are needed to confirm these results and to determine whether integrating inflammatory and cardiac biomarkers into existing risk scores improves early risk stratification.

## 5. Conclusions

In this cohort of patients with acute pulmonary embolism, ABO blood group was not associated with baseline biomarker profiles and did not materially influence the prognostic value of key laboratory markers. Instead, early cardiac strain, systemic inflammation, and renal dysfunction, captured by NT-proBNP, CRP, procalcitonin, creatinine, and D-dimer, were the principal biochemical determinants of in-hospital mortality, severe PE manifestations, and infectious or septic complications. These findings indicate that short-term prognosis in acute PE is driven primarily by the acute pathophysiological burden rather than by inherited ABO type. Incorporation of objective biomarkers of right ventricular dysfunction and inflammation may enhance early risk assessment, whereas ABO blood group does not appear to have a clinically meaningful role in prognostic evaluation.

## Figures and Tables

**Table 1:** Baseline characteristics stratified by ABO blood group.

Characteristic	O (*n* = 69)	A (*n* = 158)	B (*n* = 54)	AB (*n* = 36)
**Demographics**				
Age (years), mean ± SD	67.4 ± 15.1	68.2 ± 13.9	73.1 ± 12.7	65.4 ± 15.9
Female sex *n* (%)	38 (55.1%)	79 (50.0%)	30 (55.6%)	16 (44.4%)
Urban residence *n* (%)	46 (66.7%)	87 (55.1%)	32 (59.3%)	24 (66.7%)
**Comorbidities prior to admission *n* (%)**				
Chronic obstructive pulmonary disease	3 (4.3%)	16 (10.1%)	7 (13.0%)	2 (5.6%)
Hypertension	49 (71.0%)	107 (67.7%)	35 (64.8%)	23 (63.9%)
Atrial fibrillation	12 (17.4%)	27 (17.1%)	9 (16.7%)	4 (11.1%)
Chronic venous insufficiency	17 (24.6%)	31 (19.6%)	8 (14.8%)	1 (2.8%)
Stroke history	16 (23.2%)	21 (13.3%)	8 (14.8%)	8 (22.2%)
Asthma	2 (2.9%)	8 (5.1%)	2 (3.7%)	0 (0.0%)
Chronic kidney disease	23 (33.3%)	27 (23.4%)	19 (35.2%)	10 (27.8%)
Myocardial infarction	11 (15.9%)	8 (5.1%)	9 (16.7%)	2 (5.6%)
Brain atrophy	13 (18.8%)	18 (11.4%)	8 (14.8%)	6 (16.7%)
Dementia	7 (10.1%)	13 (8.2%)	8 (14.8%)	4 (11.1%)
Hematologic disease	25 (36.2%)	51 (32.3%)	14 (25.9%)	15 (41.7%)
Antiplatelet therapy prior to admission	10 (14.7%)	39 (24.8%)	17 (31.5%)	11 (31.4%)
Anticoagulation therapy prior to admission	25 (36.2%)	39 (26.0%)	11 (20.4%)	8 (22.2%)
Pulmonary hypertension	39 (56.5%)	85 (54.1%)	28 (51.9%)	17 (47.2%)
Diabetes	16 (23.2%)	18 (11.4%)	11 (20.4%)	5 (13.9%)
Active cancer	18 (26.1%)	34 (21.5%)	8 (14.8%)	7 (19.4%)
Obesity	23 (33.3%)	51 (32.3%)	14 (25%)	9 (25.0%)

**Table 2:** In-hospital outcomes stratified by ABO group (O, A, B, AB).

Outcome	O	A	B	AB
In-hospital mortality	10 (14.5%)	11 (7.0%)	8 (14.8%)	6 (16.7%)
Composite severe PE	21 (30.4%)	42 (26.6%)	18 (33.3%)	13 (36.1%)
Thrombolysis	2 (2.9%)	19 (12.0%)	1 (1.9%)	4 (11.1%)
CPAP	0 (0.0%)	2 (1.3%)	2 (3.7%)	1 (2.8%)
Intubation	14 (20.3%)	19 (12.0%)	12 (22.2%)	9 (25.0%)
Infection	40 (58.0%)	71 (44.9%)	25 (46.3%)	17 (47.2%)
Sepsis	10 (14.5%)	13 (8.2%)	9 (16.7%)	5 (13.9%)

**Table 3:** Median Baseline Biomarker Concentrations by ABO Blood Group.

Biomarker	O	A	B	AB
C-reactive protein (mg/dL)	44.3 (22.6–93.3)	32.8 (13.2–118.8)	47.7 (13.9–92.7)	27.7 (7.3–76.4)
CK-MB (IU/L)	21.0 (16.5–29.0)	21.5 (15.0–33.2)	21.5 (16.0–30.2)	22.0 (14.5–38.8)
Creatinine (mg/dL)	1.2 (0.9–1.3)	1.1 (0.9–1.4)	1.2 (0.9–1.4)	1.2 (0.9–1.3)
D-dimer (mg/L)	4.9 (3.1–10.8)	7.2 (3.9–13.0)	5.3 (3.6–12.1)	7.2 (3.2–12.6)
Leukocytes (×10^9^/L)	9.6 (2.4–12.1)	8.6 (5.3–11.8)	8.3 (5.0–12.5)	8.1 (2.6–11.2)
NT-proBNP (pg/mL)	1172.0 (187.5–2847.0)	1069.0 (387.0–3941.0)	859.0 (457.0–3450.0)	423.0 (170.5–4114.0)
Procalcitonin (µg/L)	0.1 (0.1–0.2)	0.1 (0.1–0.2)	0.1 (0.1–0.3)	0.1 (0.1–0.1)
Platelets (×10^9^/L)	228 (194–277)	229 (168–292)	202 (151–271)	221 (188–268)

**Table 4:** Associations Between ABO Blood Group (Non-O vs. O) and baseline biomarkers.

Biomarker	N	GMR Non-O vs. O	95% CI	*p* Value
NT-proBNP (pg/mL)	182	1.40	0.80–2.45	0.244
CK-MB (IU/L)	279	1.03	0.89–1.20	0.687
Creatinine (mg/dL)	295	0.97	0.90–1.06	0.521
C-reactive protein (mg/dL)	261	0.80	0.57–1.14	0.214
Procalcitonin (µg/L)	183	1.06	0.90–1.24	0.477
D-dimer (mg/L)	258	1.16	0.90–1.48	0.249
Leukocytes (×10^9^/L)	294	0.97	0.80–1.18	0.765
Platelets (×10^9^/L)	263	0.93	0.82–1.04	0.20

**Table 5:** Biomarkers as independent predictors of in-hospital outcomes.

Biomarker	In-Hospital Mortality OR (95% CI)	Severe PE OR (95% CI)	In-Hospital Infection OR (95% CI)	In-Hospital Sepsis OR (95% CI)
NT-proBNP	4.14 (1.90–9.01)	1.75 (1.19–2.59)	1.29 (0.92–1.81)	2.76 (1.54–4.95)
CK-MB	1.37 (0.95–1.99)	1.36 (1.05–1.78)	1.01 (0.78–1.29)	1.22 (0.84–1.78)
Creatinine	1.37 (0.99–1.89)	1.20 (0.93–1.56)	0.84 (0.62–1.13)	1.61 (1.17–2.21)
C-reactive protein	1.66 (1.02–2.71)	1.30 (0.97–1.74)	2.08 (1.55–2.79)	3.92 (2.15–7.15)
Procalcitonin	2.22 (1.39–3.52)	1.62 (1.18–2.24)	2.58 (1.61–4.12)	4.76 (2.71–8.34)
D-dimer	1.19 (0.79–1.79)	1.21 (0.91–1.60)	1.43 (1.09–1.88)	1.73 (1.17–2.55)
Leukocytes	1.49 (0.97–2.30)	0.61 (0.47–0.79)	1.41 (1.09–1.82)	1.10 (0.76–1.59)
Platelets	0.78 (0.56–1.09)	0.88 (0.69–1.14)	1.10 (0.86–1.40)	0.90 (0.64–1.24)

**Table 6:** Associations between baseline biomarkers and in-hospital mortality.

Biomarker	N	Events	OR per 1 SD (95% CI)	*p* Value
NT-proBNP (pg/mL)	181	15	4.14 (1.90–9.01)	<0.001
CK-MB (IU/L)	277	32	1.37 (0.95–1.99)	0.094
Creatinine (mg/dL)	293	34	1.37 (0.99–1.89)	0.061
C-reactive protein (mg/dL)	259	25	1.66 (1.02–2.71)	0.041
Procalcitonin (µg/L)	179	16	2.22 (1.39–3.52)	<0.001
D-dimer (mg/L)	256	27	1.19 (0.79–1.79)	0.411
Leukocytes (×10^9^/L)	292	34	1.49 (0.97–2.30)	0.068
Platelets (×10^9^/L)	291	33	0.78 (0.56–1.09)	0.148

**Table 7:** Associations between baseline biomarkers and composite severe pulmonary embolism.

Biomarker	N	Events	OR per 1 SD (95% CI)	*p* Value
NT-proBNP (pg/mL)	181	48	1.75 (1.19–2.59)	0.005
CK-MB (IU/L)	278	80	1.36 (1.05–1.78)	0.022
Creatinine (mg/dL)	294	85	1.20 (0.93–1.56)	0.159
C-reactive protein (mg/dL)	260	73	1.30 (0.97–1.74)	0.077
Procalcitonin (µg/L)	180	54	1.62 (1.18–2.24)	0.003
D-dimer (mg/L)	257	74	1.21 (0.91–1.60)	0.183
Leukocytes (×10^9^/L)	293	85	0.61 (0.47–0.79)	<0.001
Platelets (×10^9^/L)	292	84	0.88 (0.69–1.14)	0.337

**Table 8:** Associations between baseline biomarkers and in-hospital infection.

Biomarker	N	Events	OR per 1 SD (95% CI)	*p* Value
NT-proBNP (pg/mL)	181	90	1.29 (0.92–1.81)	0.135
CK-MB (IU/L)	278	137	1.01 (0.78–1.29)	0.953
Creatinine (mg/dL)	294	143	0.84 (0.62–1.13)	0.240
C-reactive protein (mg/dL)	260	132	2.08 (1.55–2.79)	<0.001
Procalcitonin (µg/L)	180	100	2.58 (1.61–4.12)	<0.001
D-dimer (mg/L)	257	128	1.43 (1.09–1.88)	0.010
Leukocytes (×10^9^/L)	293	143	1.41 (1.09–1.82)	0.008
Platelets (×10^9^/L)	292	142	1.10 (0.86–1.40)	0.452

**Table 9:** Associations between baseline biomarkers and in-hospital sepsis.

Biomarker	N	Events	OR per 1 SD (95% CI)	*p* Value
NT-proBNP (pg/mL)	181	26	2.76 (1.54–4.95)	<0.001
CK-MB (IU/L)	278	35	1.22 (0.84–1.78)	0.300
Creatinine (mg/dL)	294	37	1.61 (1.17–2.21)	0.003
C-reactive protein (mg/dL)	260	34	3.92 (2.15–7.15)	<0.001
Procalcitonin (µg/L)	180	32	4.76 (2.71–8.34)	<0.001
D-dimer (mg/L)	257	34	1.73 (1.17–2.55)	0.006
Leukocytes (×10^9^/L)	293	37	1.10 (0.76–1.59)	0.618
Platelets (×10^9^/L)	292	36	0.90 (0.64–1.24)	0.509

**Table 10:** Interaction between NT-proBNP and ABO blood group for in-hospital outcomes.

Outcome	OR per 1 SD in O (95% CI)	OR per 1 SD in Non-O (95% CI)	N	Events	*p* for Interaction
In-hospital mortality	5.47 (0.29–102.58)	3.96 (1.74–9.01)	181	15	0.63
Composite severe PE	2.69 (0.86–8.37)	1.74 (1.12–2.69)	181	48	0.429
In-hospital infection	2.33 (0.87–6.25)	1.10 (0.76–1.59)	181	90	0.034
In-hospital sepsis	18.71 (1.09–321.08)	2.54 (1.36–4.73)	181	26	0.192

## Data Availability

The data presented in this study are not publicly available due to ethical and legal restrictions related to patient privacy. Anonymized data supporting the findings of this study may be made available from the corresponding author upon reasonable request and with approval from the local ethics committee.

## Abbreviations

The following abbreviations are used in this manuscript:

A	Blood group A Digital Publishing Institute
AB	Blood group A
ABO	Blood group system (A, B, AB, O)
AF	Atrial fibrillation
ALAT	Alanine aminotransferase
APTT	Activated partial thromboplastin time
ASAT	Aspartate aminotransferase
B	Blood group
BD	Direct bilirubin
BT	Total bilirubin
CHF	Congestive heart failure
CK-MB	Creatine kinase MB isoenzyme
CI	Confidence interval
CPAP	Continuous positive airway pressure
CRP	C-reactive protein
CTEPH	Chronic thromboembolic pulmonary hypertension
CTPA	Computed tomography pulmonary angiography
DVT	Deep vein thrombosis
ESC	European Society of Cardiology
GGT	Gamma-glutamyl transferase
IQR	Interquartile range
INR	International normalized ratio
LDH	Lactate dehydrogenase
LDL-C	Low-density lipoprotein cholesterol
NT-proBNP	N-terminal pro-brain natriuretic peptide
NYHA	New York Heart Association (heart failure classification)
O	Blood group O
OR	Odds ratio
PE	Pulmonary embolism
PESI	Pulmonary Embolism Severity Index
PT	Prothrombin time
RR	Risk ratio
RV	Right ventricle/right ventricular
sPESI	Simplified Pulmonary Embolism Severity Index
SD	Standard deviation
vWF	von Willebrand factor
VSH	Erythrocyte sedimentation rate
VTE	Venous thromboembolism
